# Supplementary material for: Circulating Alpha-Tocopherol Levels, Bone Mineral Density, and Fracture: Mendelian Randomization Study
Source: Nutrients. 2021 Jun 5;13(6):1940. doi: 10.3390/nu13061940 (PMC8228419; doi:10.3390/nu13061940)
Supplement: Supplementary file 1 [file nutrients-13-01940-s001.zip › nutrients-1251714-SI.pdf]

Supplementary Table S1. Summary statistics data for the associations of the single-nucleotide polymorphisms with α-tocopherol and their associations with bone mineral density and any fracture

					Association with α-tocopherol			Association with BMD			Association with any fracture		
**SNP**	**Chr**	**Nearby gene**	**EA**	**OA**	**Beta***	**SE**	**P-value**	**Beta***	**SE**	**P-value**	**Beta***	**SE**	**P-value**
rs964184	11	BUD13/ZNF259/APOA5	G	C	0.04	0.01	7.8E-12	0.00186	0.00271	0.49	0.00376	0.00964	0.70
rs2108622	19	CYP4F2	T	C	0.03	0.01	1.4E-10	0.01279	0.00202	2.3E-10	-0.00348	0.00712	0.63
rs11057830	12	SCARB1	A	G	0.03	0.01	8.2E-9	0.01238	0.00268	3.9E-06	-0.01302	0.00949	0.17

BMD, bone mineral density; Chr, chromosome; EA, effect allele; OA, other allele; SE, standard error; SNP, single-nucleotide polymorphism.

*The beta coefficients are in log-transformed mg/L for α-tocopherol, g/cm^2 for bone mineral density, and log-odds ratios for fracture.
